# Superior knee self-efficacy and quality of life throughout the first year in patients who recover symmetrical muscle function after ACL reconstruction

**DOI:** 10.1007/s00167-019-05703-z

**Published:** 2019-09-25

**Authors:** Ramana Piussi, Susanne Beischer, Roland Thomeé, Eric Hamrin Senorski

**Affiliations:** 1 Sportrehab Sports Medicine Clinic, Stampgatan 14, 411 01 Gothenburg, Sweden; 2 Unit of Physiotherapy, Department of Health and Rehabilitation, Institute of Neuroscience and Physiology, Sahlgrenska Academy, University of Gothenburg, Box 455, 405 30 Gothenburg, Sweden

**Keywords:** Anterior cruciate ligament, Knee, Psychological outcomes

## Abstract

**Purpose:**

The aim of this study was to (1) describe psychological outcomes during the first year after an anterior cruciate ligament (ACL) reconstruction and (2) compare psychological outcomes in patients who recover symmetrical muscle function with patients who do not.

**Methods:**

The included patients had undergone a unilateral ACL reconstruction. Patients with a re-rupture and contralateral ACL injury were excluded. Three groups, based on the results from 5 tests of muscle function 12 months after reconstruction, were created. Three validated questionnaires (the Knee Self-Efficacy Scale; the Knee injury and Osteoarthritis Outcome Score subscale “Quality of Life”; the ACL Return to Sport after Injury scale) and a single question “Have you achieved your goal with rehabilitation?” were analysed in 4 different follow-ups after ACL reconstruction (10 weeks, 4, 8 and 12 months). Means and standard deviations were analysed with standard *t* tests and reported with 95% confidence intervals.

**Results:**

A total of 328 patients (120 men, 37%), mean age 27.8 ± 10 years, were included. Patients who did not recover symmetrical muscle function (*n* = 56; 17%) at the 12-month follow-up reported inferior knee-related self-efficacy and quality of life than patients who recovered symmetrical muscle function (*n* = 96; 29%) at all follow-ups, except quality of life at 4 months. The proportion of patients who stated they achieved their rehabilitation goal at 12 months was 17% for the entire cohort, 24% for patients who recovered muscle function and 5% for patients who did not recover muscle function.

**Conclusion:**

Patients who recovered strength and hop symmetry 12 months after ACL reconstruction had superior knee-related self-efficacy and greater quality of life during the whole first year after ACL reconstruction. These results can aid clinicians in the decision-making process by providing knowledge of patients who might need further attention during rehabilitation.

**Level of evidence:**

III.

**Electronic supplementary material:**

The online version of this article (10.1007/s00167-019-05703-z) contains supplementary material, which is available to authorized users.

## Introduction

An anterior cruciate ligament (ACL) rupture is a severe knee injury [12]. Like other severe injuries, patients who sustain an ACL injury may suffer from a negative psychological response, including mood disturbance, depression, increased tension, fear, anger, anxiety and reduced self-esteem [6, 25]. These psychological responses can negatively affect rehabilitation outcome after an ACL reconstruction. For instance, high fear and anxiety levels can lead to low adherence to a rehabilitation protocol, which might lead to inferior rehabilitation outcome [18]. Strong self-efficacy and low fear of re-injury are suggested to be important factors for successful rehabilitation after an ACL reconstruction [23]. Christino et al. [6] suggested that identifying patients at risk of a negative psychological response pre-operatively may help to direct post-operative interventions.

Recently, Webster et al. [35] reported that more symmetrical hop performance in athletes was associated with superior psychological readiness to return to sport. According to a biopsychosocial model related to athletic injury, both physical and functional factors affect psychological response [36, 38]. A negative psychological response comprising, for instance, depression and anxiety pre-operatively, might impact psychological readiness to return to sport after surgery. However, in the study by Webster et al. [35] only 1 hop test was used to assess limb symmetry and it is not known whether the use of more hop tests, complemented with muscle strength tests, would help to better understand the relationship between psychological readiness and muscle function. When the demands for symmetry between limbs in tests of muscle function increase by adding more tests, the success rate (that is, the number of patients reaching a given threshold for symmetry) decreases [31]. As a result, not accounting for enough hop and muscle strength tests after ACL reconstruction might create misleadingly high success rates that will jeopardise the decision-making process for return to sports.

Return to sport is a milestone for the majority of patients who sustain an ACL rupture [10]. However, only about 50% of all patients actually return to competitive sport, despite the fact that almost 90% of the patients reach the benchmarks of what is regarded as normal knee function when determined by 1 patient-reported outcome and 2 hop tests [2, 3].

There is an apparent knowledge gap with regard to psychological factors during the first post-operative year and the way these factors are related to the recovery of strength and hop ability after an ACL reconstruction. The purpose of this study was, therefore, to describe psychological outcomes during the first year after an ACL reconstruction. A further aim was to compare the psychological outcomes in patients who recover symmetrical muscle function with that of patients who do not. The hypothesis was that patient-reported psychological outcomes improve gradually during the first year of rehabilitation after ACL reconstruction. In addition, it was hypothesised that patients who recover symmetrical muscle function across a battery of tests would report superior scores for psychological outcomes compared with patients who do not recover symmetrical muscle function.

## Materials and methods

Data were extracted from a rehabilitation-specific outcome registry, Project ACL. Upon participation, patients agree to have their data collected and analysed in the registry. Follow-up data collection consists of patient-reported outcomes (PROs) and data from five tests of muscle function. Ethical approval was obtained from the Regional Ethical Review Board in Gothenburg, Sweden (registration numbers: 265-13, T023-17).

The tests of muscle function comprise knee flexion and extension strength and three tests of hop performance. The tests in Project ACL are conducted according to a standardised protocol. Patients participating in the project are recommended to familiarise themselves with all the tests prior to testing. Follow-up data are collected after a predefined schedule starting from baseline (ACL injury or reconstruction): 4 weeks, 10 weeks, 4 months, 8 months, 12 months, 18 months, 2 years, yearly up to 5 years and then every 5 years.

Patients were included in the present study if they were aged 15–65 years, had sustained a unilateral ACL injury and had undergone an ACL reconstruction. Patients were excluded if they had suffered an ACL re-rupture, a contralateral ACL injury or if they had not performed all 5 tests of muscle function at the 12-month follow-up.

### Physical outcomes

#### Muscle strength

Muscle strength was measured with a concentric isokinetic test with the patient in a seated position at 90°/s, for knee extension from 90° of flexion to full knee extension and for knee flexion from full extension to 90° flexion, using a Biodex System 4 (Biodex Medical System, Shirley, New York, USA) [32]. Strength testing with Biodex has been reported to be reliable (ICC = 0.95) when measuring muscle strength [9].

Before testing, patients started with a standardised warm-up procedure. During testing, patients were instructed to perform 3 maximum trials (in both extension and flexion). Between each trial, 30 s of rest was given. The peak torque (Nm) of the best trial for knee extension and knee flexion was used for further analysis.

#### Hop performance

Three hop tests were performed after the muscle strength tests in the following order: the single-leg vertical jump (Muscle lab, Ergotest Technology, Oslo, Norway), the single-leg hop for distance and the single-leg side hop. Each hop test was performed with the patient holding his/her hands behind his/her back. For the vertical jump and the hop for distance, patients were instructed to perform 2–5 practice trials, followed by 3 maximum trials, where the best attempt was recorded and analysed. For the single-leg side hop, patients were instructed to perform as many jumps as possible during 30 s over 2 lines, 40 centimetres apart. Three minutes of rest were given between legs and only 1 attempt per leg was allowed. The battery of hop tests has been reported to be reliable (ICC 0.95–0.97), sensitive (91%) and accurate (88%) for measuring hop performance in patients after an ACL reconstruction [16].

All the results from the muscle strength and hop tests are presented in percent as the Limb Symmetry Index (LSI), which is calculated as the ratio between the result for the injured leg divided by the result for the non-injured leg, multiplied by 100.

### Patient-reported psychological outcomes

#### Knee Self-Efficacy Scale

The Knee Self-Efficacy Scale (K-SES) aims to measure knee-related self-efficacy in patients with an ACL injury. The original scale is a 22-item score with reported good reliability (ICC = 0.75) and good validity [28]. Patients respond to each item on an 11-point Likert scale from 0 to 10, where 0 indicates poor self-efficacy and 10 indicates strong self-efficacy. The responses for each item are summarised and divided by the number of items. In this study, a shorter version (consisting of 18 items) was used. The shorter version contains 4 items fewer than the original K-SES and some items are somewhat rephrased. Reliability (ICC = 0.92), structure and validity for the short version are the same or better than the version (unpublished data). The scale aims to report present and future self-efficacy (K-SES_present_ and K-SES_future_). Data from the K-SES were analysed for the 10-week and 4-, 8- and 12-month follow-ups.

#### Knee injury and Osteoarthritis Outcome Score “Quality of Life”

The Knee injury and Osteoarthritis Outcome Score (KOOS) has five subscales: pain, symptoms, activity of daily living, function in sports and recreation and quality of life (QoL). The KOOS is both reliable (ICC = 0.83–0.95) and valid for use in patients with an ACL injury. Patients answer the questions with respect to the previous week. Standardised responses are given on a 5-point Likert scale and each answer has a value ranging from 0 to 4. A normalised score from 0 to 100 is calculated for each subscale, where 0 indicates the most severe symptoms and 100 indicates no symptoms. In the present study, the subscale of QoL (4 items) was used [27]. Data on the KOOS QoL were analysed for the 10-week and 4-, 8- and 12-month follow-ups.

#### ACL Return to Sport after Injury scale

The ACL Return to Sport after Injury scale (ACL-RSI) is reliable (Cronbach’s *α* = 0.95) and valid to assess psychological readiness to return to sport. The ACL-RSI has a fair to good ability to predict return to sport [34, 35]. In this study, the 12-item version was used. The scale is graded from 0 to 10, where 0 means an extremely negative psychological response and 10 an extremely positive one [33, 34]. Data from the ACL-RSI were analysed for the 8- and 12-month follow-ups.

#### Single question: “have you achieved your goal with rehabilitation?”

In this study, patients were asked to answer a single question, “have you achieved your goal with rehabilitation?” (yes/no). Data for the single question were analysed for the 10-week and 4-, 8- and 12-month follow-ups.

A recent systematic review [13] identified ACL-RSI as the psychological PRO with the highest methodological quality, and KOOS as the most commonly used PRO for patients with an ACL injury, and was, therefore, used in this study. The K-SES is the only knee-specific PRO reflecting self-efficacy in patients with an ACL injury and was, therefore, used to reflect the psychological outcome of self-efficacy of knee function in the present study.

### Definition of study groups

Patients were divided into 3 groups based on the results for muscle function recovery (LSI) at 12 months. According to suggested consensus criteria [24] for a successful muscle function outcome after ACL injury or reconstruction, muscle strength measured with the LSI, in the present study, was set to be at least 90%, whereas a value below 85% was regarded as an unsuccessful outcome. Accordingly, the first muscle function recovery group was defined as patients with a high LSI (H LSI) and consisted of patients who reached symmetrical muscle function with an LSI of ≥ 90% in all 5 muscle function tests. The second group was defined as patients with a low LSI (L LSI) and consisted of patients who did not recover symmetrical muscle function and had an LSI of 85% or lower in at least 1 strength test and 1 hop test. The third group was defined as patients with LSI values between H LSI and L LSI, referred to as mid-LSI (M LSI). Based on demographic differences, analysis was performed first between groups and second between groups divided by sex.

### Statistical analysis

Means, medians, standard deviations (SD) and ranges were reported for patient demographics and outcomes. Comparisons of the means for each of the PROs were performed between the 3 groups using a standardised *t* test and presented with 95% confidence intervals [4]. For comparison of median values (Tegner Activity Scale), a Mann–Whitney *U* test was used. When comparing the proportion of patients who reported achieving their goal with rehabilitation across the 3 LSI groups, a Chi square test was used [4]. For the single question about achieving the goal of rehabilitation, comparisons were only performed for the 12-month follow-up, as a limited number of patients achieved their goal at the 4- and 8-month follow-ups. Effect sizes (ES) (Cohen’s *D*) were calculated for all comparisons. An effect size between 0.20 and 0.50 is defined as small, between 0.51 and 0.80 as medium, and 0.81 and above as large [22].

Post hoc power analyses were performed with an online-based clinical calculator (https://clincalc.com/stats/Power.aspx) for all comparisons between LSI groups.

## Results

### Study sample

**Fig. 1:**
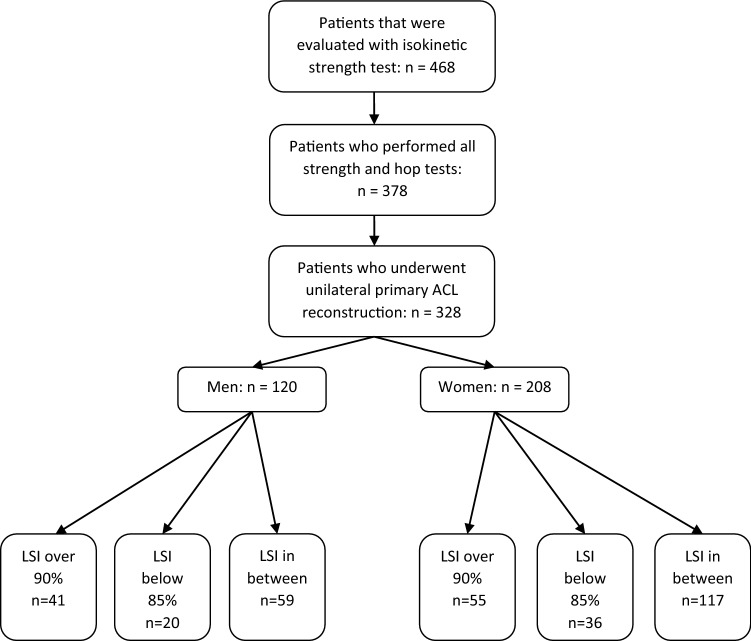
Flowchart of inclusion and exclusion of the study population. *ACL* anterior cruciate ligament, *LSI* Leg Symmetry Index, *n* number

A total of 328 patients (120 men and 208 women) were included in this study (Fig. 1). Up to June 2018, 468 patients were evaluated for strength and hop performance in Project ACL, of which 378 (80%) completed all the strength and hop tests at the 12-month follow-up. There were 50 patients (13%) who were excluded because they had sustained an ACL re-rupture or a contralateral ACL rupture. The included patients were 26 (± 9.9) years of age (range 15-65) on average at the time of reconstruction. The majority of patients (83%) underwent ACL reconstruction with a hamstring graft and the mean time between injury and reconstruction was 373 days (range 6–7483) (Table 1).

**Table 1:** Patient demographics and comparison between sexes

	All (*n* = 328)		Men (*n* = 120)		Women (*n* = 208)		*p* values
	Mean (SD)	Range	Mean (SD)	Range	Mean (SD)	Range	
Age at reconstruction (years)	26 (9.9)	15–64	28.7 (9.6)	15–59	24.5 (9.7)	15–65	< 0.001*
Height (cm)	173.8 (9.3)	150–209	180.7 (6.4)	163–200	169.8 (8.4)	150–209	< 0.001*
Weight (kg)	71.3 (12.4)	45–111	79.9 (10.2)	55–111	66.3 (10.8)	45–107	< 0.001*
BMI (kg/m^2^)	23.3 (2.8)	18–33	24.4 (2.5)	19–33.2	22.7 (2.8)	17.6–32.9	< 0.001*
Pre-injury Tegner (median)	8	1–10	8	1–10	8	2–10	n.s.
Hamstring graft *N* (%)	273 (83.2%)		96 (80%)		177 (85.1%)		n.s.
Patella graft *N* (%)	45 (13.7%)		20 (16.7%)		25 (12%)		n.s.
Allograft *N* (%)	4 (1.2%)		1 (0.8%)		3 (1.4%)		
Other graft *N* (%)	2 (0.6%)		0		2 (1%)		
Missing graft data *N* (%)	4 (1.2%)		3 (2.5%)		1 (0.5%)		
Days between injury and reconstruction	373 (832.3)	6–7483	359 (734.7)	7–5685	381.1 (885.4)	6–7483	n.s.
Outcomes at 12-month follow-up							
LSI quadriceps	95.8 (11.3)	54–198	96.7 (9.8)	70–123	95.2 (12.1)	54–198	n.s.
LSI hamstring	98.4 (13.4)	8–207	97.7 (10.6)	74–128	98.8 (14.8)	8–207	n.s.
LSI vertical hop	90.3 (15.7)	49–149	91.6 (15.8)	49–149	89.5 (15.7)	50–149	n.s.
LSI distance hop	94.5 (9.4)	48–119	95.3 (10.6)	52–119	94.1 (8.7)	48–115	n.s.
LSI side hop	95.9 (18.2)	25–171	97.7 (17.1)	47–167	94.9 (18.8)	25–171	n.s.

*BMI* body mass index, *cm* centimetres, *kg* kilograms, *LSI* Limb Symmetry Index, *n* number, *Tegner* Tegner Activity Scale, *SD* standard deviation

*Statistically significant difference

The 3 groups that were created, based on the muscle function results at 12 months after ACL reconstruction, consisted of 96 patients in the H LSI group, 56 patients in the L LSI group and 176 patients in the M LSI group (Table 2).

**Table 2:** Patient demographics and comparison between the Limb Symmetry Index groups

	H LSI (*n* = 96)	L LSI (*n* = 56)	M LSI (*n* = 176)	*p* value 1 (H vs L)	*p* value 2 (H vs M)	*p* value 3 (L vs M)
Women *n* (%)	55 (57%)	36 (64%)	117 (66%)			
Age (years)	25.9 (10.3)	30.3 (10.3)	24.7 (9.1)	0.012*	n.s.	< 0.001*
Height (cm)	175.2 (10.9)	173.5 (8.5)	173.1 (8.6)	n.s.	n.s.	n.s.
Weight (kg)	73 (13.5)	72.1 (11.9)	70.2 (11.9)	n.s.	n.s.	n.s.
BMI (kg/m^2^)	23.6 (2.5)	23.8 (2.5)	23.1 (3)	n.s.	n.s.	n.s.
Pre-injury Tegner (median; min–max)	8 (3–10)	8 (4–10)	8 (1–10)	n.s.	n.s.	n.s.
Hamstring graft *n* (%)	79 (82%)	47 (83%)	147 (83%)			
Patella graft *n* (%)	11 (11%)	8 (14%)	26 (14%)			
Allograft *n* (%)	1 (1%)	2 (3%)	1 (0.5%)			
Other graft *n* (%)	1 (1%)	0	1 (0.5%)			
Missing graft data *n* (%)	3 (3%)	0	1 (0.5%)			
Days between injury and reconstruction	572.9 (1302.7)	206 (322)	317.1 (553.7)	0.010*	n.s.	n.s.

*BMI* body mass index, *cm* centimetres, *H LSI* group with high LSI, *kg* kilograms, *M LSI* group with LSI in between, *L LSI* group with low LSI, *LSI* Limb Symmetry Index, *n* number, *Tegner* Tegner Activity Scale, *SD* standard deviation

*Statistically significant difference

### K-SES present and future

**Fig. 2:**
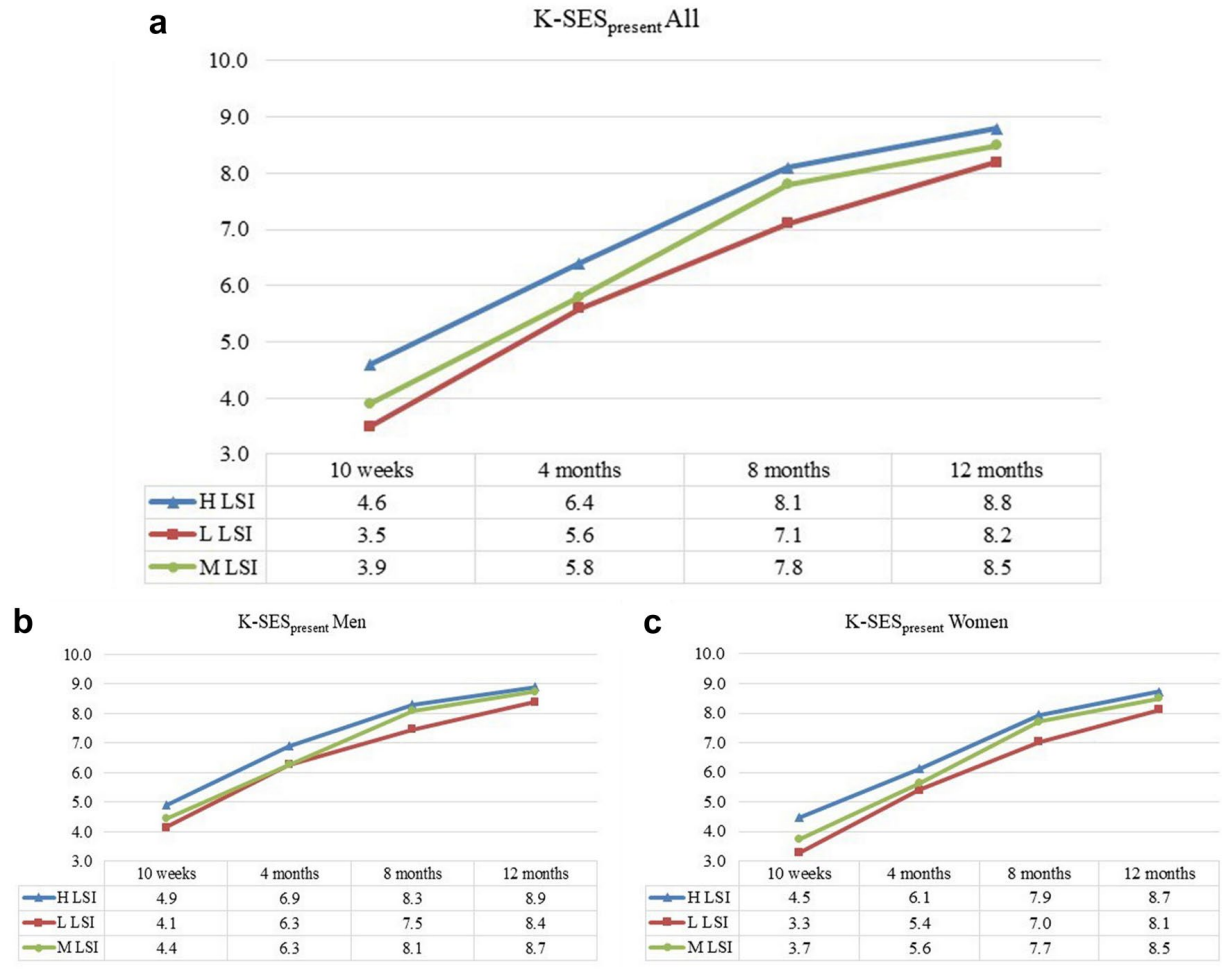
The Knee Self-Efficacy Scale (K-SES) subscale present presented over time. **a** (All subjects), **b** (men) and **c** (women). **a** The results for the K-SES_present_ for all subjects, divided into H LSI (blue), L LSI (red) and M LSI (green). **b**, **c** The results for men and women, respectively, divided into the three LSI groups

Patients in the H LSI group reported a higher K-SES_present_ compared with patients in the L LSI group at each follow-up (10 weeks, *p* = 0.002, ES 0.59; 4 months, *p* = 0.027, ES 0.44; 8 months, *p* < 0.001, ES 0.63; and 12 months, *p* = 0.003, ES 0.51) (Fig. 2). In addition, patients in the H LSI group reported a higher K-SES_present_ than patients in the M LSI group at each follow-up, except at 8 months (10 weeks, *p* = 0.010, ES 0.37; 4 months *p* = 0.009, ES 0.35; 8 months *p* = n.s. and 12 months *p* = 0.049, ES 0.24). When comparing the K-SES_present_ between the M LSI and L LSI groups, patients in the M LSI group reported a higher K-SES_present_ at the 8-month (*p* = 0.004, ES 0.47) and 12-month (*p* = 0.05, ES 0.30) follow-ups.

**Fig. 3:**
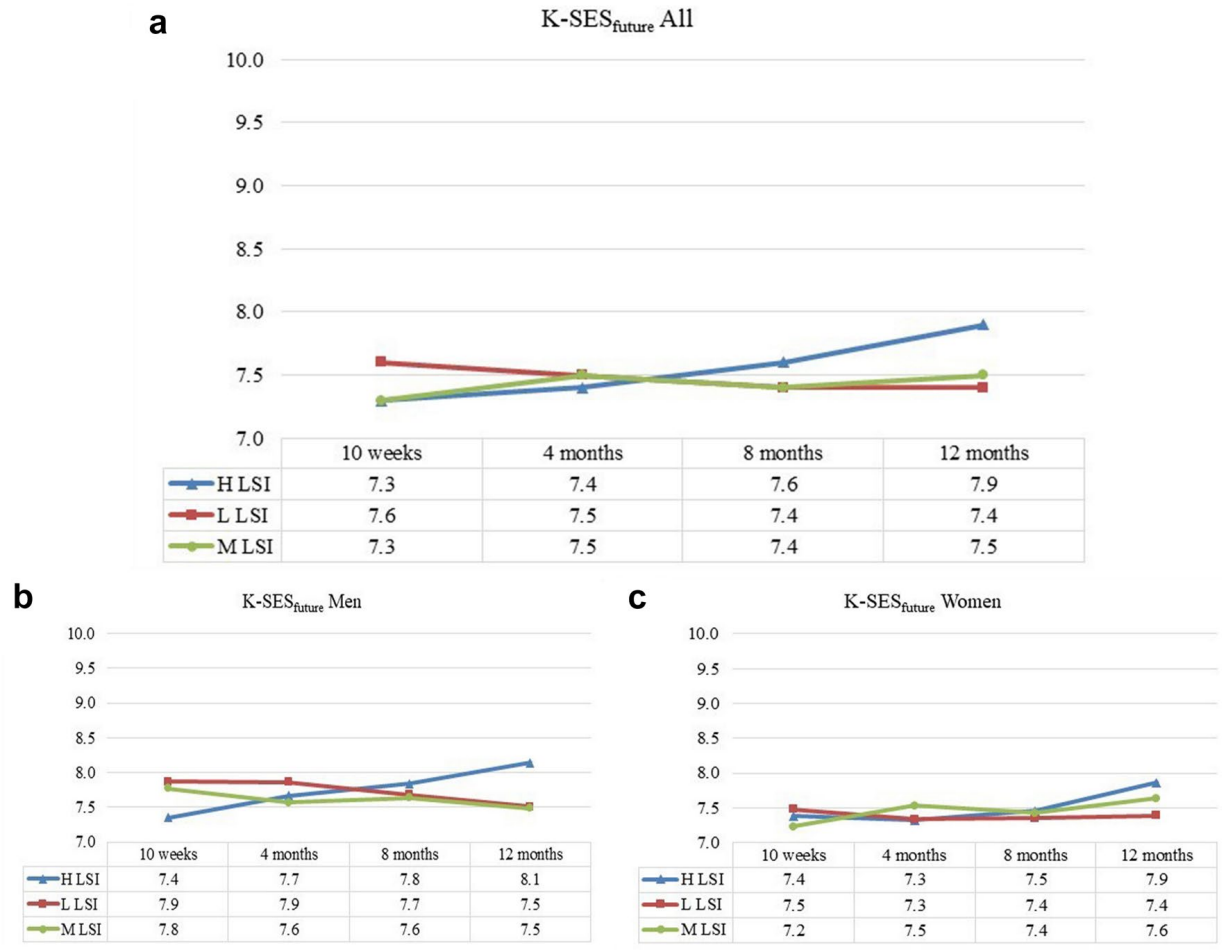
The Knee Self-Efficacy Scale (K-SES) subscale future presented over time. **a** (All subjects), **b** (men) and **c** (women). **a** The results for the K-SES_future_ for all subjects, divided into LSI (blue), L LSI (red) and M LSI (green). **b**, **c** The results for men and women, respectively, divided into the three LSI groups

For the K-SES_future_, there were no statistically significant differences between the LSI groups at any of the follow-ups (Fig. 3).

### KOOS QoL

**Fig. 4:**
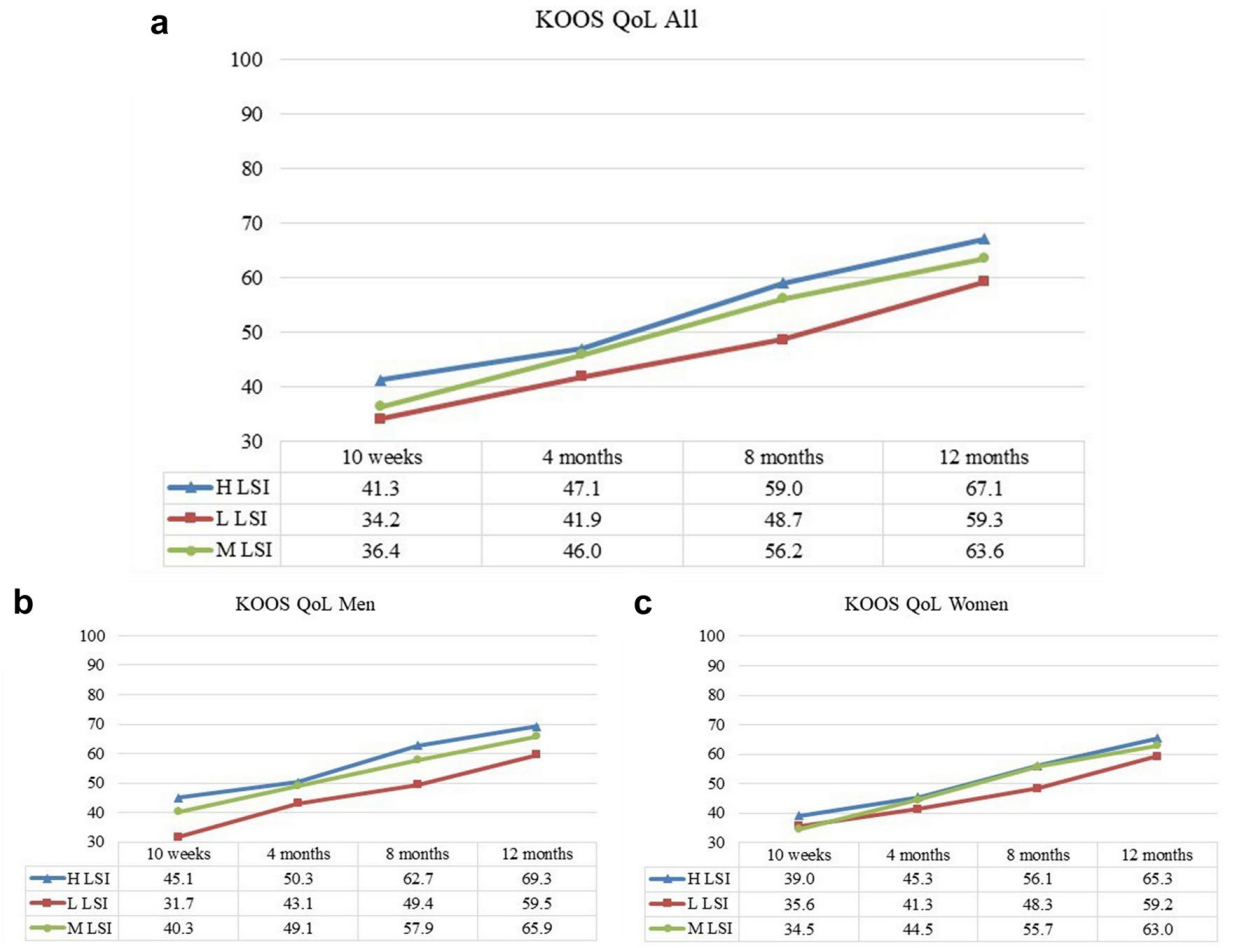
The Knee injury and Osteoarthritis Outcome Score (KOOS) subscale Quality of Life (QoL) presented over time. **a** (All subjects), **b** (men) and **c** (women). **a** The results for the KOOS QoL for all subjects, divided into H LSI (blue), L LSI (red) and M LSI (green). **b**, **c** The results for men and women, respectively, divided into the three LSI groups

Patients in the H LSI group reported a higher KOOS QoL compared with patients in the L LSI group 10 weeks (*p* = 0.012, ES 0.47), 8 months (*p* = 0.001, ES 0.56) and 12 months (*p* = 0.007, ES 0.46) after ACL reconstruction. When compared with the M LSI group, patients in the H LSI group reported a higher KOOS QoL (41.3 versus 36.4 points, *p* = 0.036, ES 0.31) at the 10-week follow-up after ACL reconstruction. At 8 months, patients in the M LSI group reported 56.2 points versus 48.7 points in the L LSI group (*p* = 0.006, ES 0.43) (Fig. 4).

### ACL-RSI

**Fig. 5:**
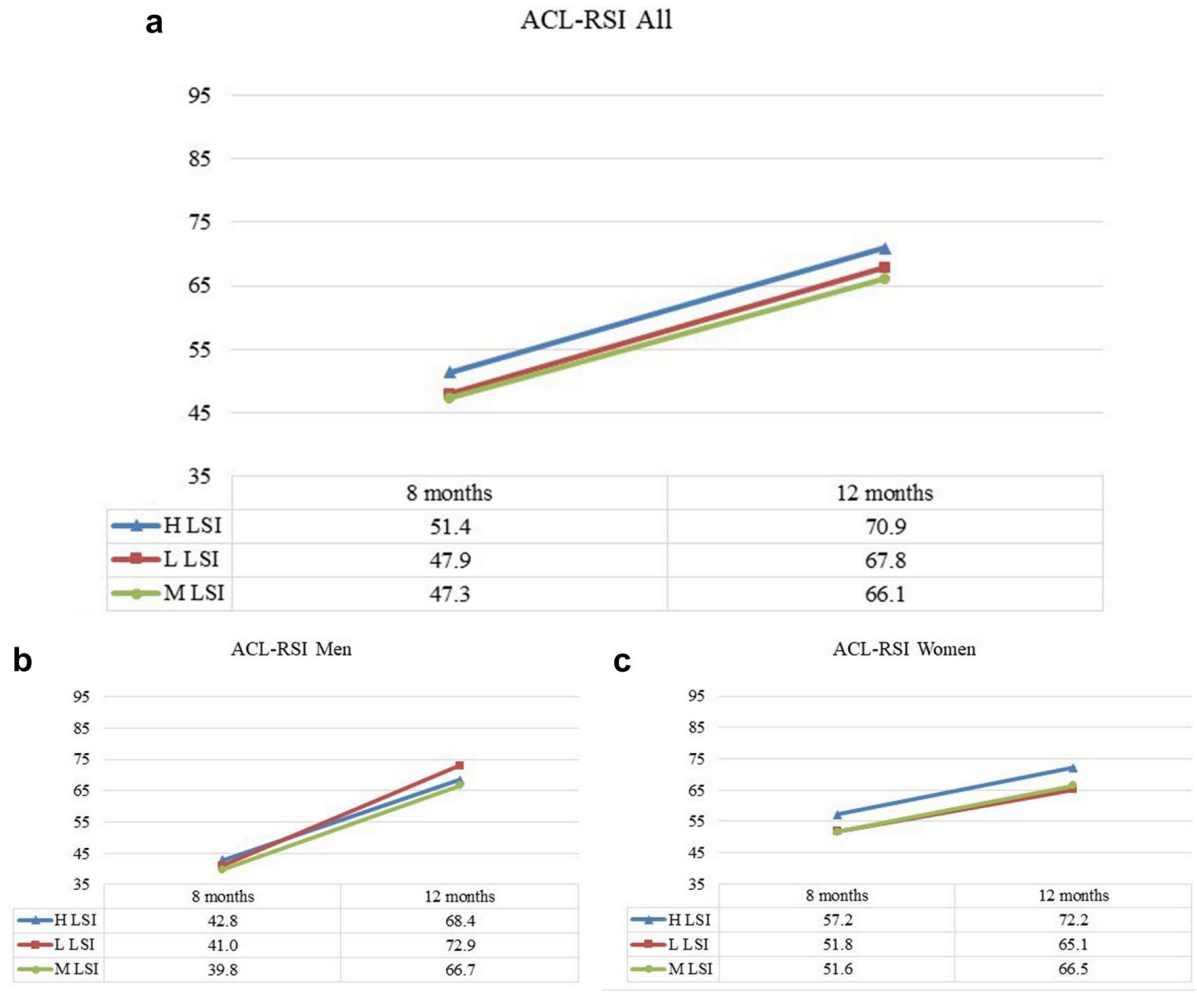
The ACL Return to Sport after Injury Scale (ACL-RSI) presented over time. **a** (All subjects), **b** (men) and **c** (women). **a** The results for the ACL-RSI for all subjects, divided into H LSI (blue), L LSI (red) and M LSI (green). **b**, **c** The results for men and women, respectively, divided into the three LSI groups.

For the ACL-RSI, there were no differences between the LSI groups at any of the follow-ups, although the psychological readiness to return to sport changed for all the groups between 8 and 12 months (Fig. 5).

### Achieving the individual goal of rehabilitation

When asked whether patients had achieved their goal with rehabilitation, 2% of the patients answered “Yes” at 8 months and 17% at 12 months after ACL reconstruction (Table 3).

**Table 3:** The proportion of patients having reported to achieve their goal of rehabilitation during 12 months after ACL reconstruction

	Yes/no *n* (%)
10 weeks	1/227 (0.004)
4 months	1/261 (0.003)
8 months	7/291 (2.4)
12 months	50/295 (17)

*n* number

**Fig. 6:**
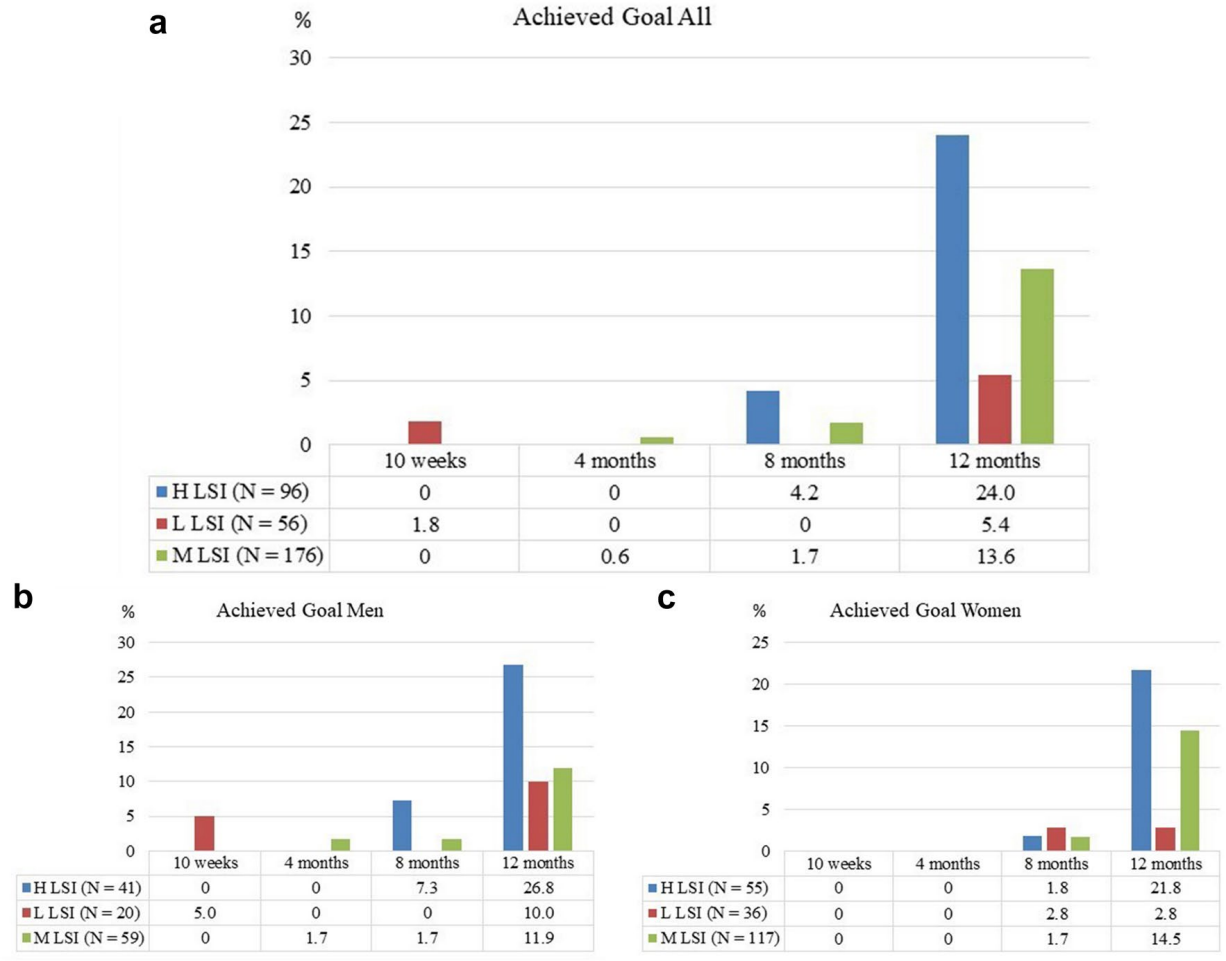
Reponses to the single question “Have you achieved your goal with your rehabilitation?” presented over time. **a** The proportion of patients answering “yes”, for H LSI (blue), L LSI (red) and M LSI (green). **b**, **c** The proportion of men and women, respectively, achieving their goals with rehabilitation

The proportions of patients who had achieved their goal at 12 months were 24% in the H LSI group, 5% in the L LSI group (*p* = 0.003 when compared with the H LSI group) and 13% in the M LSI group (*p* = 0.014 when compared with the H LSI group) (Fig. 6).

The significant differences in the results from the comparisons made in this study, and the effect sizes (ES) for the significant differences are summarised in Table 4.

**Table 4:** Summary of results for group comparisons

	All			Men			Women		
	H LSI L LSI	H LSI M LSI	M LSI LSI L	H LSI L LSI	H LSI M LSI	M LSI L LSI	H LSI L LSI	H LSI M LSI	M LSI L LSI
K-SES present									
10 weeks	● 0.59	● 0.37					● 0.64	● 0.40	
4 months	● 0.44	● 0.35			● 0.40				
8 months	● 0.63		● 0.47	● 0.65			● 0.65		● 0.49
12 months	● 0.51	● 0.24	● 0.30				● 0.52		
K-SES future									
10 weeks									
4 months									
8 months									
12 months									
KOOS Qol									
10 weeks	● 0.47	● 0.31		● 0.83					
4 months									
8 months	● 0.56		● 0.43	● 0.77			● 0.45		● 0.41
12 months	● 0.46								
ACL RSI									
8 months									
12 months									
Have you achieved your goal?									
10 weeks									
4 months									
8 months									
12 months	●	●					●	●	

Each dot indicates a significant difference (*p* < 0.05). For each significant difference, the effect size is reported

ACL RSI = Anterior Cruciate Ligament Return to Sport  after Injury Scale; K-SES = Knee Self-Efficacy Score; LSI = limb symmetry index; H LSI = group with high LSI; L LSI = group with low LSI; M LSI = group with LSI in between; QoL = Quality of Life (KOOS subscale)

## Discussion

The main finding in this study was that patients who had recovered their muscle function in a battery of 5 muscle function tests 12 months after ACL reconstruction reported superior present self-efficacy and quality of life throughout the first post-operative year, compared with patients who had not recovered their muscle function. However, fewer than 1 in 5 patients reported having achieved their goal with rehabilitation 12 months after ACL reconstruction. When the patients were divided into groups based on their LSI from the tests of muscle function, 24% of the patients in the High LSI group (H LSI, ≥ 90% in LSI in the 5 tests) reported that they had achieved their goal, compared with only 5% in the Low LSI group (L LSI, < 85% in LSI in at least 1 strength and 1 hop test). This study gives clinicians an insight into how the psychological outcomes may vary during rehabilitation after ACL reconstruction. In particular, the outcomes for K-SES_present_ and KOOS QoL appear to be useful to follow patients’ psychological progress during rehabilitation, as both outcomes were able to identify differences at 12 months between patients who recovered muscle function and patients who did not.

The fact that fewer than 1 in 5 patients achieve their goal 1 year after ACL reconstruction indicates that 1 year should not be seen as a time-based cutoff for resuming pre-injury activity and that rehabilitation can take longer. From the results in the present study, it cannot be determined how recovery of muscle strength is associated with psychological outcomes. However, in the model described by Wiese Björnstahl [38], the recovery process is dynamic, and the predominant direction of recovery is that cognitive appraisal affects emotions, which in turn affect behaviours [38]. According to this model, a patient with a stronger psychological profile after ACL reconstruction can behave in a way that leads to accomplish rehabilitation goals, e.g. attend every visit and put maximal effort into training, to a greater extent compared with a patient with a weaker psychological profile. Therefore, low psychological outcomes early after ACL reconstruction can help to identify the patients who will struggle to recover muscle function 1 year after ACL reconstruction. Regular assessments of psychological outcomes during rehabilitation can aid physiotherapists in directing extra resources (e.g. additional and longer sessions) to patients at risk of not recovering muscle strength.

The LSI is one of the most commonly used methods to report the results from tests of muscle function. However, the LSI has recently been criticised, as it tends to overestimate the patient’s function [37]. Greater symmetry between limbs in a battery of tests has, however, been reported to reduce the risk of ACL re-rupture [15]. An LSI level of ≥ 90% is suggested as symmetrical and sufficient when it is realised across different muscle function tests. Recently, a battery consisting of 7 isokinetic strength and hop tests (Back In Action-BIA) was evaluated and 1 in 40 (2.5%) patients managed to recover symmetry through all tests [8]. This suggests that even 5 tests, as used in the present study, might underestimate the patient’s muscle function.

In the present study, the K-SES_present_ resulted in several statistical differences. However, there is uncertainty regarding knee self-efficacy and what difference is a minimal clinically relevant difference for patients after ACL reconstruction. A more than one unit difference is suggested as a relevant difference in the K-SES outcome [29]. In the present study, the K-SES_present_ was compared between the 3 LSI groups at all follow-ups for a total of 36 comparisons, of which almost half were significant (*p* < 0.05). However, in only 2 comparisons (H LSI versus L LSI at 10 weeks and 8 months) was the difference greater than one unit. The results must, therefore, be interpreted with caution. The K-SES appears to potentially have prognostic value, as patients with higher levels of self-efficacy have been reported to have superior outcomes in terms of return to sport or level of physical activity, less impairment and greater satisfaction during ACL rehabilitation [1, 5, 17, 30]. On the other hand, the K-SES_future_ subscale provided little clinical value in the present study, as no difference was found when analysing future knee self-efficacy. Moreover, no clear progression over time, or relationship with muscle function was observed for the K-SES_future_. It could, however, be argued that a stable belief in one’s future knee-related self-efficacy could be positive. As the K-SES_future_ results do not change over time, it could indicate that, despite better or worse present muscle function, patients believe they will be able to trust their knee in the future.

Lower levels of KOOS QoL have been associated with several negative outcomes after ACL reconstruction, such as not returning to the same level of knee-related activity [17], greater fear of re-injury [21] and ACL revision [14]. Interventions that improve knee-related quality of life in these patients should be addressed during rehabilitation. Unfortunately, it is not known how best to address knee-related quality of life during rehabilitation. A recent systematic review reported inconsistent findings when psychological interventions (relaxation and guided imagery) were added to the rehabilitation to improve KOOS QoL and reduce fear of re-injury and anxiety after an ACL reconstruction [7]. The results were, however, only based on 4 studies. Despite the inconsistent findings for relaxation and guided imagery [7], other psychological intervention methods have been associated with improving health-related problems. Mindfulness and cognitive-behavioural therapy (CBT) have been shown to improve physical functioning, chronic pain, depression, anxiety, low self-esteem [19, 20, 39]. However, there appears to be a need for a tool which aims to improve quality of life, as it is still unclear whether targeting muscle function may help to increase patients’ perception of quality of life. Despite a promising relationship between muscle function and patient-reported quality of life, the results in this study do not provide an answer to the relationship between muscle function and quality of life.

Muller et al. [26] suggested a threshold of 62.5 points on the KOOS QoL as the cutoff between an acceptable state of “feeling well” and not “feeling well” in patients 1–6 years after ACL reconstruction. According to this patient-acceptable cutoff, patients in the H LSI and M LSI groups reached an acceptable state at 12 months on average, while patients in the L LSI group did not. This raises the question of whether patients who fail at least 1 strength and 1 hop test can reach a patient-acceptable QoL before improving their muscle function, or whether these patients will benefit from merely being given more time for rehabilitation. However, it is possible that the progression in KOOS QoL outcome for patients struggling to recover muscle function may reach a plateau, which is supported by findings reported by Filbay et al. [11], who described a 20-year follow-up of QoL after ACL reconstruction, where low QoL was found 5–25 years after reconstruction in ACL-injured patients compared with normative data.

The ACL-RSI has been developed during the past decade [33, 34]. In the present study, there was no significant difference in ACL-RSI between patients who recovered their muscle function symmetry 12 months after ACL reconstruction and patients who did not. The present study design is unable to determine the factors that influence this relationship. The majority of patients were probably determined to return to sport after their ACL reconstruction, but only 17% of the patients stated that they had achieved their goal. Hypothetically, if the majority of patients had a return to sport as a rehabilitation goal, this finding may explain the small differences in the ACL-RSI, which aims to measure psychological readiness to return to sport.

Statistical analysis generated 8 comparisons per group and PRO (24 when stratified by sex). A major limitation in this study is the risk of type 1 error, due to the many comparisons. Only one of the differences in this study (KOOS QoL for men, H LSI versus L LSI at 10 weeks) had a large effect size (0.83). All the other effect sizes varied between 0.24 and 0.77, entailing that the results should be interpreted with caution.

Another limitation of this study was that each follow-up consisted of a cross-sectional cohort of patients. However, the proportion of patients in each group who had responded at each follow-up (Online Appendix Table 3) was never below 51% and most commonly above 80%. Another limitation was that comparisons of groups stratified by sex might have led to few patients in certain groups and underpowered results. However, before stratifying by sex, the groups consisted of 96, 56 and 176 patients, respectively, which led to comparisons reaching statistical power (above 80%).

## Conclusion

Patients who recovered strength and hop symmetry 12 months after ACL reconstruction had superior present knee-related self-efficacy and higher quality of life during the whole first year after reconstruction. These results can aid clinicians in the decision-making process by providing knowledge of patients who might need further attention during rehabilitation.

## Electronic supplementary material

Below is the link to the electronic supplementary material.

Supplementary material 1 (DOCX 25 kb)

## References

[1] Ardern CL, Osterberg A, Sonesson S, Gauffin H, Webster KE, Kvist J (2016). Satisfaction with knee function after primary anterior cruciate ligament reconstruction is associated with self-efficacy, quality of life, and returning to the preinjury physical activity. Arthroscopy.

[2] Ardern CL, Taylor NF, Feller JA, Webster KE (2014). Fifty-five per cent return to competitive sport following anterior cruciate ligament reconstruction surgery: an updated systematic review and meta-analysis including aspects of physical functioning and contextual factors. Br J Sports Med.

[3] Ardern CL, Taylor NF, Feller JA, Whitehead TS, Webster KE (2015). Sports participation 2 years after anterior cruciate ligament reconstruction in athletes who had not returned to sport at 1 year: a prospective follow-up of physical function and psychological factors in 122 athletes. Am J Sports Med.

[4] Björk J (2011) Praktisk statistik för medicin och hälsa. Liber

[5] Chmielewski TL, George SZ (2018). Fear avoidance and self-efficacy at 4 weeks after ACL reconstruction are associated with early impairment resolution and readiness for advanced rehabilitation. Knee Surg Sports Traumatol Arthrosc.

[6] Christino MA, Fantry AJ, Vopat BG (2015). Psychological aspects of recovery following anterior cruciate ligament reconstruction. J Am Acad Orthop Surg.

[7] Coronado RA, Bird ML, Van Hoy EE, Huston LJ, Spindler KP, Archer KR (2018). Do psychosocial interventions improve rehabilitation outcomes after anterior cruciate ligament reconstruction? A systematic review. Clin Rehabil.

[8] Ebert JR, Edwards P, Currie J, Smith A, Joss B, Ackland T (2018). Comparison of the ‘Back In Action’ test battery to standard hop tests and isokinetic knee dynamometry in patients following anterior cruciate ligament reconstruction. Int J Sports Phys Ther.

[9] Feiring DC, Ellenbecker TS, Derscheid GL (1990). Test–retest reliability of the biodex isokinetic dynamometer. J Orthop Sports Phys Ther.

[10] Feucht MJ, Cotic M, Saier T, Minzlaff P, Plath JE, Imhoff AB (2016). Patient expectations of primary and revision anterior cruciate ligament reconstruction. Knee Surg Sports Traumatol Arthrosc.

[11] Filbay SR (2018). Longer-term quality of life following ACL injury and reconstruction. Br J Sports Med.

[12] Forssblad M (2016) Svenska Korsbandsregister årsrapport. https://aclregister.nu/info/rapport2016.pdf. Accessed 18 May 2018

[13] Gagnier JJ, Shen Y, Huang H (2018). Psychometric properties of patient-reported outcome measures for use in patients with anterior cruciate ligament injuries: a systematic review. JBJS Rev.

[14] Granan LP, Baste V, Engebretsen L, Inacio MC (2015). Associations between inadequate knee function detected by KOOS and prospective graft failure in an anterior cruciate ligament-reconstructed knee. Knee Surg Sports Traumatol Arthrosc.

[15] Grindem H, Snyder-Mackler L, Moksnes H, Engebretsen L, Risberg MA (2016). Simple decision rules can reduce reinjury risk by 84% after ACL reconstruction: the Delaware–Oslo ACL cohort study. Br J Sports Med.

[16] Gustavsson A, Neeter C, Thomee P, Silbernagel KG, Augustsson J, Thomee R (2006). A test battery for evaluating hop performance in patients with an ACL injury and patients who have undergone ACL reconstruction. Knee Surg Sports Traumatol Arthrosc.

[17] Hamrin Senorski E, Samuelsson K, Thomee C, Beischer S, Karlsson J, Thomee R (2017). Return to knee-strenuous sport after anterior cruciate ligament reconstruction: a report from a rehabilitation outcome registry of patient characteristics. Knee Surg Sports Traumatol Arthrosc.

[18] Ivarsson A, Tranaeus U, Johnson U, Stenling A (2017). Negative psychological responses of injury and rehabilitation adherence effects on return to play in competitive athletes: a systematic review and meta-analysis. Open Access J Sports Med.

[19] Khoo EL, Small R, Cheng W, Hatchard T, Glynn B, Rice DB (2019). Comparative evaluation of group-based mindfulness-based stress reduction and cognitive behavioural therapy for the treatment and management of chronic pain: a systematic review and network meta-analysis. Evid Based Ment Health.

[20] Kolubinski DC, Frings D, Nikcevic AV, Lawrence JA, Spada MM (2018). A systematic review and meta-analysis of CBT interventions based on the Fennell model of low self-esteem. Psychiatry Res.

[21] Kvist J, Ek A, Sporrstedt K, Good L (2005). Fear of re-injury: a hindrance for returning to sports after anterior cruciate ligament reconstruction. Knee Surg Sports Traumatol Arthrosc.

[22] Lakens D (2013). Calculating and reporting effect sizes to facilitate cumulative science: a practical primer for t-tests and ANOVAs. Front Psychol.

[23] Lentz TA, Paterno MV, Riboh JC (2018). So you think you can return to sport?. Br J Sports Med.

[24] Lynch AD, Logerstedt DS, Grindem H, Eitzen I, Hicks GE, Axe MJ (2015). Consensus criteria for defining ‘successful outcome’ after ACL injury and reconstruction: a Delaware–Oslo ACL cohort investigation. Br J Sports Med.

[25] Morrey MA, Stuart MJ, Smith AM, Wiese-Bjornstal DM (1999). A longitudinal examination of athletes’ emotional and cognitive responses to anterior cruciate ligament injury. Clin J Sport Med.

[26] Muller B, Yabroudi MA, Lynch A, Lai CL, van Dijk CN, Fu FH (2016). Defining thresholds for the patient acceptable symptom state for the IKDC subjective knee form and KOOS for patients who underwent ACL reconstruction. Am J Sports Med.

[27] Roos EM, Lohmander LS (2003). The knee injury and osteoarthritis outcome score (KOOS): from joint injury to osteoarthritis. Health Qual Life Outcomes.

[28] Thomee P, Wahrborg P, Borjesson M, Thomee R, Eriksson BI, Karlsson J (2006). A new instrument for measuring self-efficacy in patients with an anterior cruciate ligament injury. Scand J Med Sci Sports.

[29] Thomee P, Wahrborg P, Borjesson M, Thomee R, Eriksson BI, Karlsson J (2007). Self-efficacy, symptoms and physical activity in patients with an anterior cruciate ligament injury: a prospective study. Scand J Med Sci Sports.

[30] Thomeé P, Währborg P, Börjesson M, Thomeé R, Eriksson B, Karlsson J (2008). Self-efficacy of knee function as a pre-operative predictor of outcome 1 year after anterior cruciate ligament reconstruction. Knee Surg Sports Traumatol Arthrosc.

[31] Thomee R, Neeter C, Gustavsson A, Thomee P, Augustsson J, Eriksson B (2012). Variability in leg muscle power and hop performance after anterior cruciate ligament reconstruction. Knee Surg Sports Traumatol Arthrosc.

[32] Undheim MB, Cosgrave C, King E, Strike S, Marshall B, Falvey E (2015). Isokinetic muscle strength and readiness to return to sport following anterior cruciate ligament reconstruction: is there an association? A systematic review and a protocol recommendation. Br J Sports Med.

[33] Webster KE, Feller JA (2018). Development and validation of a short version of the anterior cruciate ligament return to sport after injury (ACL-RSI) scale. Orthop J Sports Med.

[34] Webster KE, Feller JA, Lambros C (2008). Development and preliminary validation of a scale to measure the psychological impact of returning to sport following anterior cruciate ligament reconstruction surgery. Phys Ther Sport.

[35] Webster KE, Nagelli CV, Hewett TE, Feller JA (2018). Factors associated with psychological readiness to return to sport after anterior cruciate ligament reconstruction surgery. Am J Sports Med.

[36] Weiss MR (2003). Psychological aspects of sport-injury rehabilitation: a developmental perspective. J Athl Train.

[37] Wellsandt E, Failla MJ, Snyder-Mackler L (2017). Limb symmetry indexes can overestimate knee function after anterior cruciate ligament injury. J Orthop Sports Phys Ther.

[38] Wiese-Bjornstal DM, Smith AM, Shaffer SM, Morrey MA (1998). An integrated model of response to sport injury: psychological and sociological dynamics. J Appl Sport Psychol.

[39] Zhang A, Franklin C, Jing S, Bornheimer LA, Hai AH, Himle JA (2019). The effectiveness of four empirically supported psychotherapies for primary care depression and anxiety: a systematic review and meta-analysis. J Affect Disord.

