# Supplementary material for: Superior knee self-efficacy and quality of life throughout the first year in patients who recover symmetrical muscle function after ACL reconstruction
Source: Knee Surg Sports Traumatol Arthrosc. 2019 Sep 25;28(2):555–67. doi: 10.1007/s00167-019-05703-z (PMC6994536; doi:10.1007/s00167-019-05703-z)
Supplement: Supplementary file 1 — Supplementary material 1 (DOCX 25 kb) [file 167_2019_5703_MOESM1_ESM.docx]

**Appendix**

| Appendix Table 1: Patient demographics stratified by sex and by Limb Symmetry Index group. | | | | | | | | |
| --- | --- | --- | --- | --- | --- | --- | --- | --- |
|  | | Men | | | | Women | | |
|  | H LSI  (n = 41) | | L LSI  (n = 20) | M LSI  (n = 59) | H LSI  (n = 55) | | L LSI  (n = 36) | M LSI  (n = 117) |
| Age (years) | 28.5 (10.8) | | 32.4 (11.8) | 27.6 (7.8) | 23.9 (9.7) | | 29.2 (9.4) | 23.2 (9.4) |
| Height (cm) | 181.6 (7.9) | | 180.3 (5.3) | 180.3 (5.7) | 169.8 (8.4) | | 169.8 (7.7) | 169.5 (7.6) |
| Weight (kg) | 79.6 (11.4) | | 79.9 (9.4) | 80.2 (9.8) | 66.3 (10.8) | | 67.8 (11.1) | 65.1 (9.6) |
| BMI | 24.1 (2.2) | | 24.6 (2.5) | 24.6 (2.6) | 23.2 (2.7) | | 23.3 (2.4) | 22.3 (3) |
| Pre-injury Tegner  (median) | 8 (4-10) | | 7 (3-10) | 8 (1-10) | 8 (3-10) | | 8 (4-10) | 8 (2-10) |
| Hamstring graft n (%) | 32 (80.5%) | | 14 (70%) | 49 (83.1%) | 46 (83.6%) | | 33 (91.7%) | 98 (83.8%) |
| Patella graft n (%) | 5 (12.2%) | | 6 (30%) | 9 (15.3%) | 6 (10.9%) | | 2 (5.6%) | 17 (14.5%) |
| Allograft n (%) | 1 (2.4%) | | 0 | 0 | 1 (1.8%) | | 1 (2.8%) | 1 (0.9%) |
| Other graft n (%) | 0 | | 0 | 0 | 1 (1.8%) | | 0 | 1 (0.9%) |
| Missing graft data n (%) | 2 (4.9%) | | 0 | 1 (1.7%) | 1 (1.8%) | | 0 | 0 |
| Days between injury and reconstruction | 543.2 (995.4) | | 149.2 (99.5) | 302.1 (615.2) | 595 (1500) | | 237.6 (394) | 324.6 (522.6) |
| BMI = Body Mass Index; cm = centimetres; H LSI = group with high LSI; kg = kilograms; M LSI = group with LSI in between; L LSI = group with low LSI; LSI = Limb Symmetry Index; n = number; Tegner = Tegner Activity Scale. | | | | | | | | |

| Appendix Table 2: Mean (Standard Deviation) Limb Symmetry Index from tests om muscle function stratified by group. | | | | | | |  |
| --- | --- | --- | --- | --- | --- | --- | --- |
|  | High Group (N = 96) | Low Group (N = 56) | Mid Group (N = 176) | P value (high vs low) | P value (high vs INB) | P value (INB vs low) | |
| LSI quadriceps | 100.9 (7.2) | 84.1 (9.7) | 96.7 (11.1) | <0.001* | 0.001* | <0.001* | |
| LSI hamstring | 102.7 (8.5) | 89 (16) | 99 (13.4) | <0.001* | 0.014* | <0.001* | |
| LSI vertical hop | 102.7 (11) | 74.6 (13.3) | 88.5 (13.2) | <0.001* | <0.001* | <0.001* | |
| LSI distance hop | 100. (5.7) | 86.3 (12.1) | 94.2 (8) | <0.001* | <0.001* | <0.001* | |
| LSI sidehop | 105.9 (11.9) | 82.1 (17.6) | 94.9 (18.1) | <0.001* | <0.001* | <0.001* | |
| N = number; LSI = Leg Symmetry Index; * = statistically significant difference. | | | | | | | |

| Appendix Table 3: Patient response frequencies for patient-reported outcomes at follow-ups. Number of individuals (%). | | | | | | | |
| --- | --- | --- | --- | --- | --- | --- | --- |
|  |  |  | | |  | | |
|  |  | Men | | | Women | | |
|  |  | High | Low | In between | High | Low | In between |
| 10 weeks | K SES present | 27 (67%) | 16 (80%) | 47 (78%) | 44 (80%) | 28 (77%) | 91 (77%) |
|  | K SES future | 27 (67%) | 16 (80%) | 47 (78%) | 44 (80%) | 28 (77%) | 91 (77%) |
|  | KOOS Qol | 27 (67%) | 16 (80%) | 47 (78%) | 44 (80%) | 28 (77%) | 90 (76%) |
|  | achieved goal? | 21 (51%) | 15 (75%) | 39 (66%) | 42 (76%) | 27 (75%) | 83 (71%) |
|  |  |  |  |  |  |  |  |
| 4 months | K SES present | 31 (77%) | 16 (80%) | 51 (85%) | 49 (89%) | 31 (86%) | 99 (84%) |
|  | K SES future | 31 (77%) | 16 (80%) | 51 (85%) | 49 (89%) | 31 (86%) | 99 (84%) |
|  | KOOS Qol | 31 (77%) | 16 (80%) | 51 (85%) | 49 (89%) | 31 (86%) | 99 (84%) |
|  | achieved goal? | 27 (66%) | 14 (70%) | 44 (75%) | 49 (89%) | 31 (86%) | 96 (82%) |
|  |  |  |  |  |  |  |  |
| 8 months | K SES present | 35 (87%) | 17 (85%) | 53 (88%) | 50 (90%) | 34 (94%) | 104 (88%) |
|  | K SES future | 35 (87%) | 17 (85%) | 53 (88%) | 50 (90%) | 34 (94%) | 104 (88%) |
|  | KOOS Qol | 35 (87%) | 17 (85%) | 53 (88%) | 50 (90%) | 34 (94%) | 103 (88%) |
|  | achieved goal? | 36 (88%) | 16 (80%) | 51 (86%) | 50 (90%) | 33 (92%) | 105 (90%) |
|  | ACL-RSI | 41 (100%) | 20 (100%) | 60 (100%) | 55 (100%) | 36 (100%) | 116 (99%) |
|  |  |  |  |  |  |  |  |
| 12 months | K SES present | 39 (97%) | 19 (95%) | 58 (96%) | 51 (92%) | 34 (94%) | 109 (93%) |
|  | K SES future | 39 (97%) | 19 (95%) | 58 (96%) | 51 (92%) | 34 (94%) | 109 (93%) |
|  | KOOS Qol | 39 (97%) | 19 (95%) | 58 (96%) | 51 (92%) | 34 (94%) | 109 (93%) |
|  | achieved goal? | 36 (88%) | 17 (85%) | 57 (97%) | 47 (86%) | 32 (89%) | 106 (90%) |
|  | ACL-RSI | 41 (100%) | 20 (100%) | 60 (100%) | 55 (100%) | 36 (100%) | 116 (99%) |
| ACL-RSI = ACL Return to Sport after Injury Scale; K-SES = Knee Self Efficacy Scale; KOOS = Knee Injury and Osteoarthritis Outcome Score; Qol = Quality of Life (KOOS subscale). | | | | | | | |

| Appendix Table 4: Differences between mean values for each comparison. | | | | | | | | | | |
| --- | --- | --- | --- | --- | --- | --- | --- | --- | --- | --- |
|  |  | All | | | Men | | | Women | | |
|  |  | H-L | H-M | M-L | H-L | H-M | M-L | H-L | H-M | M-L |
| K ses now | 10 w | **1.1*** | 0.7* | 0.4 | 0.8 | 0.5 | 0.3 | **1.2*** | 0.7* | 0.5 |
|  | 4 m | 0.8* | 0.6* | 0.2 | 0.6 | 0.6* | 0.0 | 0.7 | 0.5 | 0.2 |
|  | 8 m | **1.0*** | 0.3 | 0.7* | 0.8* | 0.2 | 0.6 | 0.9* | 0.2 | 0.7* |
|  | 12 m | 0.6* | 0.3* | 0.3* | 0.5 | 0.2 | 0.4 | 0.6* | 0.2 | 0.4 |
|  |  |  |  |  |  |  |  |  |  |  |
| K ses future | 10 w | -0.3 | 0.0 | -0.3 | -0.5 | -0.4 | -0.1 | -0.1 | 0.1 | -0.3 |
|  | 4 m | -0.1 | -0.1 | 0.0 | -0.2 | 0.1 | -0.3 | 0.0 | -0.2 | 0.2 |
|  | 8 m | 0.2 | 0.2 | 0.0 | 0.2 | 0.2 | 0.0 | 0.1 | 0.0 | 0.1 |
|  | 12 m | 0.5 | 0.4 | 0.1 | 0.6 | 0.7 | 0.0 | 0.5 | 0.2 | 0.3 |
|  |  |  |  |  |  |  |  |  |  |  |
| KOOS QoL | 10 w | 7.1* | 4.9* | 2.2 | 13.4* | 4.8 | 8.6 | 3.4 | 4.5 | -1.1 |
|  | 4 m | 5.2 | 1.1 | 4.1 | 7.2 | 1.2 | 6 | 4.0 | 0.8 | 3.2 |
|  | 8 m | 10.3* | 2.8 | 7.5* | 13.3* | 4.8 | 8.5 | 7.8* | 0.4 | 7.4* |
|  | 12 m | 7.8* | 3.5 | 4.3 | 9.8 | 3.4 | 6.4 | 6.1 | 2.3 | 3.8 |
|  |  |  |  |  |  |  |  |  |  |  |
| ACL-RSI | 8 m | 3.5 | 4.1 | -0.6 | 1.8 | 3 | -1.2 | 5.4 | 5.6 | -0.2 |
|  | 12 m | 3.1 | 4.8 | -1.7 | -4.5 | 1.7 | -6.2 | 7.1 | 5.7 | 1.4 |
| ACL RSI = Anterior Cruciate Ligament Return to Sport Index; K SES = Knee Self Efficacy Scale; H = group with high LSI; L = group with low LSI; M = group with LSI in between; QoL = Quality of Life (KOOS subscale); *= statistical significance; underline = power above 80%; bold text = reached Minimal Important Change. 10w, 4m, 8m, 12m = follow-ups in the present study (10 weeks, 4 months, 8 months, 12 months) | | | | | | | | | | |

In appendix Table 4, all numbers presented are the actual differences (subtraction of mean value of one group from mean value of another group) between the listed groups. Asterisks (*) show where the differences were statistical significant, underline text shows where differences had a power above 80%, and bold text shows where differences reached minimal important changes.
